# Dual impact of flow diverters on ischemic and hemorrhagic outcomes in vertebral artery dissecting aneurysms presenting with ischemia

**DOI:** 10.3389/fneur.2026.1815552

**Published:** 2026-05-08

**Authors:** Yuto Shingai, Hiroyuki Sakata, Shunsuke Omodaka, Tomohisa Ishida, Ryosuke Tashiro, Yoshimichi Sato, Hidenori Endo

**Affiliations:** 1 Department of Neurosurgery, Kohnan Hospital, Sendai, Miyagi, Japan; 2 Department of Neuroendovascular Therapy, Kohnan Hospital, Sendai, Miyagi, Japan; 3 Department of Neurosurgery, Tohoku University Graduate School of Medicine, Sendai, Miyagi, Japan; 4 Department of Translational Neuroscience, Tohoku University Graduate School of Medicine, Sendai, Miyagi, Japan

**Keywords:** endovascular treatment, flow diversion, ischemic stroke, partially thrombosed aneurysm, subarachnoid hemorrage, vertebral artery dissecting aneurysm

## Abstract

**Introduction:**

Intracranial vertebral artery dissecting aneurysms (VADA) may manifest as ischemic stroke. Although some individuals stabilize with conservative management, aneurysmal enlargement and recurrent embolic events may still occur, especially in partially thrombosed lesions. The role of flow diversion (FD) in ischemia-onset VADA remains unclear. This study reports the first focused experience evaluating FD in VADA with ischemic onset.

**Methods:**

Among 119 FD procedures performed between 2022 and 2025, four male individuals were treated for ischemia-onset VADA. All lesions were in the V4 segment, and three showed partial thrombosis. FD was selected for aneurysmal enlargement (four individuals) or recurrent infarction (two individuals). Dual antiplatelet therapy was started at least 7 days before the procedure.

**Results:**

FD placement was technically successful in all cases, with no complications. Six-month angiography showed O’Kelly–Marotta grade C occlusion in all lesions, indicating effective flow remodeling. No individuals experienced recurrent cerebral infarction or aneurysmal rupture.

**Conclusion:**

Flow diversion may be a feasible treatment option in selected cases of ischemia-onset VADA. It may reduce the risk of recurrent embolic events and aneurysmal rupture.

## Introduction

Vertebral artery dissection with ischemic onset typically manifests in two morphological patterns: a stenotic type and an aneurysmal type. The stenotic type generally follows a favorable clinical course and is most often managed medically or conservatively (1). In contrast, the aneurysmal type, referred to as intracranial vertebral artery dissecting aneurysm (VADA), carries a higher risk because it may lead to subarachnoid hemorrhage from rupture or recurrent posterior circulation infarction when a partially thrombosed aneurysm serves as an embolic source.

For hemorrhagic-onset VADA, surgical intervention is commonly used (1), whereas medical therapy remains the first-line approach for ischemia-onset cases (2). Nonetheless, some individuals experience aneurysmal enlargement or recurrent ischemic events during follow-up, necessitating surgical consideration. Available options include deconstructive approaches, parent artery trapping with or without bypass, and reconstructive strategies such as stent-assisted coil embolization (SAC) or flow diversion (FD), with the choice guided by vascular anatomy and collateral circulation. However, robust evidence and standardized indications for these techniques remain limited, and the optimal peri-procedural antiplatelet regimen is still undefined.

Here, we report four individuals with ischemia-onset VADA treated with FD after careful evaluation of recurrent infarction and aneurysmal enlargement. To our knowledge, no prior studies have specifically examined the efficacy of FD in this population. This series provides the first description suggesting that FD may represent a reasonable therapeutic option in this clinical setting.

## Materials and methods

This study adhered to the principles of the Declaration of Helsinki and was approved by the local Institutional Review Board (approval number: 2023-0118-03). Reporting followed the Strengthening the Reporting of Observational Studies in Epidemiology (STROBE) guidelines. The requirement for informed consent was waived because the analysis used anonymized clinical data collected after each individual agreed to treatment and provided written consent.

### Patient cohort

This observational cohort study analyzed prospectively collected data from consecutive individuals with cerebral aneurysms who underwent FD treatment at our institution between July 2022 and July 2025 (*n* = 119). Within this cohort, we identified individuals with VADA with ischemic presentation and evaluated their clinical and imaging outcomes.

### Measurement of aneurysms

The diameter of the flow lumen was measured on pre-treatment digital subtraction angiography (DSA). The overall aneurysm size, including the thrombosed component, was evaluated using original image of time-of-flight MRA or three-dimensional T1-weighted variable flip-angle turbo spin-echo imaging (T1-CUBE), which enables visualization of the aneurysm wall and intramural hematoma.

### Peri-procedural management

At least 7 days before FD treatment, all individuals started dual antiplatelet therapy (DAPT), aspirin 100 mg/day plus either prasugrel 3.75 mg/day or clopidogrel 75 mg/day. DAPT was continued for 6 months after FD, followed by single antiplatelet therapy (aspirin 100 mg/day or prasugrel 3.75 mg/day) for at least another 6 months.

### Procedures

FD stent deployment was performed under general anesthesia using a triaxial system. The device was positioned to fully cover the dissecting aneurysmal segment with sufficient proximal and distal landing in the parent artery. Systemic heparinization was used to maintain the activated coagulation time within 250–300 s.

### Endpoints

Scheduled angiographic follow-up with digital subtraction angiography (DSA) was performed at 6 and 12 months after FD treatment. Aneurysm occlusion or remodeling was graded using the O’Kelly–Marotta (OKM) scale; “adequate occlusion” was defined as OKM C–D and “complete occlusion” as OKM D. Neurological outcome was assessed with the modified Rankin Scale (mRS). Clinical events of interest included peri-procedural or delayed ischemic or hemorrhagic complications.

## Results

### Patient characteristics

Among 119 FD procedures performed at our center, four male individuals treated for ischemia-onset VADA were included in this study (Table 1). All lesions were located in the V4 segment.

**Table 1:** Baseline characteristics of the study cohort.

Case	Age	Sex	Side	Dissection site	Relation to PICA	Contralateral VA	Infarction site	Mechanism	Thrombosed AN	Recurrent infarction and site	Aneurysmal enlargement
1	48	M	R	V4	involved	−	cerebellum	PICA embolism or branch occlusion	partially thrombosed	−	+
2	59	M	R	V4	distal	+	medulla	Perforator embolism or branch occlusion	partially thrombosed	−	+
3	61	M	L	V4	distal	+	Multiple lesion in pons	embolism	partially thrombosed	+ pons	+
4	57	M	R	V4	proximal	+	occipital lobe cerebellum thalamus	embolism	none	+ occipital lobe thalamus	+

PICA, posterior inferior cerebellar artery; VA, vertebral artery; AN, aneurysm.

The mechanism of infarction was considered predominantly embolic in two patients (case 3, 4), based on lesion distribution and anatomical considerations. In Case 3, the initial infarction showed bilateral multiple lesions in the upper pons, basilar artery perforator territory, and the recurrent infarction also occurred in the middle pons. The dissection was localized to the left V4 segment and did not extend to the basilar artery. Given the absence of direct basilar artery involvement, these findings were considered more consistent with an embolic mechanism rather than perforator occlusion directly related to the dissection. As an embolic source, thrombosed aneurysm was most suspicious.

In case 4, the dissection was also localized to the right V4 segment and the initial infarction involved multiple territories, including the occipital lobe, cerebellum, and thalamus. The recurrent infarctions were also observed in the occipital lobe and thalamus, further supporting an embolic origin. Also, the lesion was a vertebral artery dissection with a patent false lumen, demonstrating dilatation of both the true and false lumens. Thrombus formation may have occurred within the false lumen or at the interface between the true and false lumen, serving as a potential source of distal embolism, even in the absence of a clearly visible intra-aneurysmal thrombus.

In the remaining two patients, infarction patterns were more suggestive of branch occlusion related to the dissection; however, an embolic mechanism could not be definitively excluded. Overall, these findings indicate that ischemic mechanisms in this series were heterogeneous, with both embolic and branch-related processes potentially involved (Table 1).

For evaluation of other embolic sources, all patients underwent Holter electrocardiography and transthoracic echocardiography. No cardiogenic embolic sources were identified.

### Perioperative antithrombotic therapy

All patients had received aspirin 100 mg/day plus either prasugrel 3.75 mg/day or clopidogrel 75 mg/day at least 7 days before procedure. The cases who experienced recurrent stroke preoperatively, they received antithrombic agents after first onset. In Case 3, the patient was receiving aspirin (100 mg) following prior coronary stenting at the time of the initial infarction. Prasugrel (3.75 mg) was subsequently added, and dual antiplatelet therapy was continued for 3 weeks. After de-escalation to aspirin monotherapy, recurrent infarction occurred 7 months later, prompting re-initiation of prasugrel and further evaluation and surgical treatment. In Case 4, aspirin (100 mg) was initiated at the referring hospital after the initial infarction. No additional antiplatelet therapy was introduced after the recurrence of ischemia there. The patient was referred to our institution, and prasugrel (3.75 mg) was started one week prior to treatment.

### Intervention

The time from onset to FD treatment ranged from 1 to 84 months (median, 7 months); one individual was treated at 84 months, and the remaining three within 12 months. Indications for FD were aneurysmal enlargement and/or recurrent cerebral infarction (Tables 1, 2).

**Table 2:** Intervention and clinical outcomes.

Case	Indication for intervention	Onset to intervention (month)	Device			ΔmRS	OKM grade
			Stent	Diameter (mm)	Length (mm)		
1	Aneurysmal enlargement	4	FRED	5.5	22	0	C3 (6M)
2	Aneurysmal enlargement	84	FRED	4	32	0	C3 (1Y)
3	Aneurysmal enlargement and recurrent infarction	9	Pipeline Shield	4	25	0	C3 (6M)
4	Aneurysmal enlargement and recurrent infarction	1	Pipeline Shield	4.25	30	0	C3 (6M)

mRS, modified Rankin Scale; OKM grade, O’Kelly-Marotta grade; PICA, posterior inferior cerebellar artery; FRED, Flow Re-Direction Endoluminal Device.

Two individuals were treated with the Flow Re-Direction Endoluminal Device (FRED; MicroVention, Aliso Viejo, CA, USA), and two with the Pipeline Flex Shield (Medtronic Neurovascular, Irvine, CA, USA). All devices were deployed within the V4 segment; two spanned the V3–V4 junction, and two remained confined to V4. In three cases, the posterior inferior cerebellar artery (PICA) origin was intentionally jailed by the device. All procedures were completed without intraoperative complications, and no new diffusion-positive lesions were detected postoperatively (Table 2).

### Clinical outcomes

No individual experienced rupture or recurrent infarction after FD treatment. All four lesions achieved at least O’Kelly–Marotta (OKM) grade C at 6-month DSA follow-up. Neurologically, the modified Rankin Scale remained unchanged in all individuals during follow-up (Table 2).

### Aneurysmal characteristics

Detailed aneurysm characteristics and changes are summarized in Table 3, including the maximum diameter of the flow lumen and the overall aneurysm size incorporating the thrombosed component. All cases demonstrated aneurysmal enlargement prior to treatment, including rapid interval growth in one case. Following flow diverter placement, all aneurysms showed stabilization or shrinking without further enlargement during follow-up. No progression of brainstem compression or new brainstem edema was observed on follow-up imaging in any case.

**Table 3:** Aneurysm characteristics.

Case	Max flow lumen (mm)	Max aneurysm diameter (mm)	Pretreatment change	Post-FD change	Mass effect for brain
1	7.4	17.4	15.0 mm → 17.4 mm (2 months, +16%)	Stable (2 years)	—
2	6.3	16.3	12.3 mm → 16.3 mm (7 years, +33%)	Shrink (1 year, 14.1 mm)	—
3	5.7	12.7	6.1 mm → 12.7 mm (3 years, +108%)	Stable (1 year)	—
4	7.1	7.1	4.7 mm → 7.1 mm (2 weeks, +51%)	Stable (6 months)	—

### Case presentations

#### Case 1

**Figure 1:**
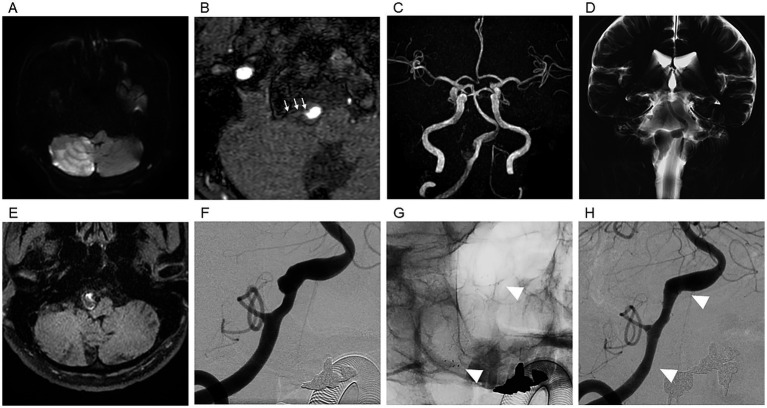
Representative case 1 (48-year-old man). **(A)** DWI on admission showing acute infarction in the right PICA territory. **(B)** TOF-MRA demonstrate that the posterior inferior cerebellar artery (PICA) origin was poorly visualized on initial imaging, which limited precise determination of the occlusion site. **(C)** TOF-MRA demonstrating right V4 dilatation. **(D)** BPAS showing outer-diameter enlargement. **(E)** 3D T1-weighted variable flip-angle TSE (T1-CUBE) image showing intraluminal thrombus (white arrow), supporting the diagnosis of a partially thrombosed dissecting aneurysm. **(F)** Preoperative DSA showing a dissecting aneurysm involving the PICA origin. **(G)** Fluoroscopic image after FRED placement (arrowheads). **(H)** Follow-up DSA at 6 months demonstrating adequate occlusion/remodeling (OKM C3). White arrowheads indicate the distal and proximal edges of the flow diverter stent.

A 48-year-old man presented to a local emergency department with occipital headache, vertigo, and vomiting. MRI revealed an acute infarction in the right PICA territory (Figure 1A). MRA suggested a right vertebral artery dissection in the V4 segment (Figure 1C) and the posterior inferior cerebellar artery (PICA) origin was poorly visualized on initial imaging, which limited precise determination of the occlusion site (Figure 1B). Basiparallel anatomical scanning (BPAS) demonstrated outer-diameter enlargement (Figure 1D). A 3D T1-weighted variable flip-angle TSE (T1-CUBE) image showed intraluminal thrombus, supporting the diagnosis of a partially thrombosed VADA (Figure 1E). The individual was referred to our hospital for surgical evaluation. DSA confirmed a VADA involving the PICA origin, with a maximal diameter of 6.4 mm. Irregular wall changes and tandem stenosis extended from the PICA origin to the vertebral artery union (Figure 1F). At this time, the PICA was clearly visualized on DSA.

The mechanism of infarction was not definitively determined. Both embolic occlusion of the PICA and branch occlusion at the PICA origin related to the dissection were considered possible. The partially thrombosed sac was possibly considered the embolic source of the PICA territory infarction, and the aneurysm enlargement was observed during outpatient follow-up, FD treatment was scheduled 4 months after onset to prevent delayed hemorrhage and recurrent embolic events.

DAPT with aspirin (100 mg/day) and clopidogrel (75 mg/day) was initiated 2 weeks before the procedure. A FRED 5.5 × 22 mm device was deployed in the right V4 segment, fully covering the thrombosed aneurysm and intentionally jailing the PICA origin (Figure 1F).

Distal dilatation of the vertebral artery was observed beyond the dissecting aneurysmal segment, from which the anterior spinal artery (ASA) originated. The vessel diameter at this level was 7.0 mm, making adequate wall apposition of the flow diverter at this site technically challenging.

A relatively normal-appearing segment with mild stenosis (inner diameter 5.4 mm) was identified proximal to the ASA origin and was selected as the distal landing zone. Although this did not represent a strict normal-to-normal deployment, the stent was placed to sufficiently cover the thrombosed portion of the aneurysm while ensuring stable wall apposition.

Post-dilation was unnecessary because cone-beam CT confirmed satisfactory wall apposition. The postoperative course was uneventful, with no new diffusion-positive lesions. Follow-up DSA at 6 months demonstrated adequate remodeling, classified as OKM grade C3, with preserved PICA flow (Figure 1G).

#### Case 2

**Figure 2:**
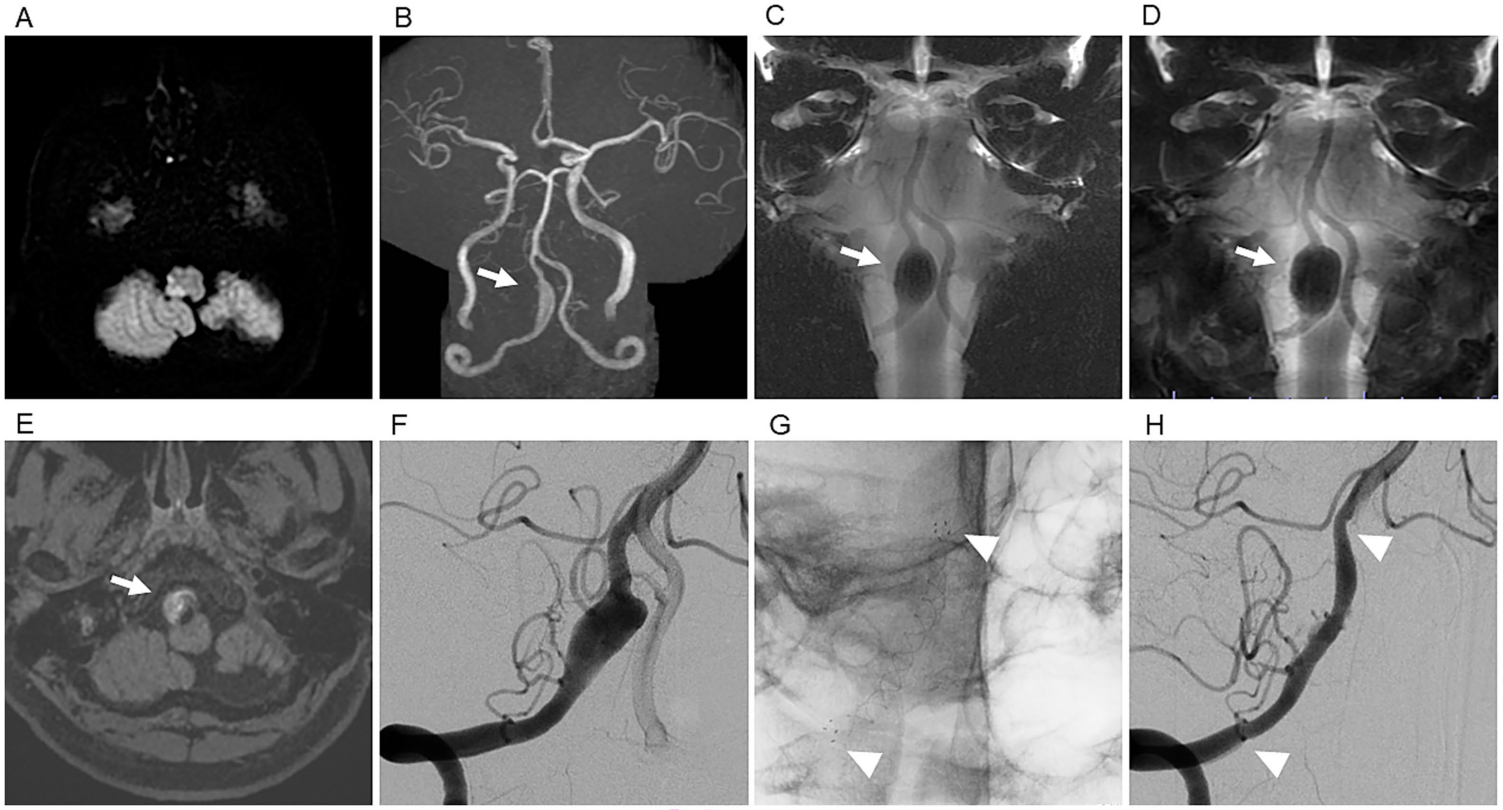
Representative case 2 (59-year-old man). **(A)** DWI on admission showing a right lateral medullary infarction. **(B)** TOF-MRA demonstrating right V4 dilatation. **(C)** BPAS showing outer-diameter enlargement, consistent with a partially thrombosed aneurysm. **(D)** BPAS obtained 7 years after initial onset showing enlargement of the aneurysm’s outer diameter. **(E)** 3D T1-weighted variable flip-angle TSE (T1-CUBE) image showing intraluminal thrombus (white arrow). **(F)** Preoperative DSA showing a dissecting aneurysm distal to the PICA origin. **(G)** Fluoroscopic image after FRED placement (arrowheads) across the V3–V4 junction. **(H)** Follow-up DSA 1 year after intervention demonstrating OKM C3 remodeling with maintained branch patency. White arrowheads indicate the distal and proximal edges of the flow diverter stent.

A 59-year-old man initially presented with neck pain and dysphagia. MRI revealed a right lateral medullary infarction (Figure 2A). MRA demonstrated dilatation of the right V4 segment (Figure 2B), and BPAS showed outer-diameter enlargement (Figure 2C). His symptoms improved with conservative management, including blood pressure control, and he was followed as an outpatient. The infarction pattern was suggestive of a perforator occlusion or embolic mechanism.

Seven years after the initial onset, follow-up MRI revealed significant aneurysm enlargement (Figure 2D). A 3D T1-weighted variable flip-angle TSE (T1-CUBE) image showed intraluminal thrombus, supporting the diagnosis of a partially thrombosed VADA (Figure 2E). The absence of recurrent ischemic events over a long interval further supports the possibility that the initial ischemia resulted from localized branch occlusion associated with the dissecting process (Table 1). The individual was treated with aspirin (100 mg/day) and prasugrel (3.75 mg/day) for 2 weeks before intervention. Preprocedural DSA demonstrated dilatation of the right vertebral artery distal to the PICA origin, with a maximal diameter of 8.3 mm (Figure 2F). A FRED 4.0 × 32 mm device was deployed to fully cover the lesion (Figure 2G). Post-dilation was unnecessary because cone-beam CT confirmed satisfactory wall apposition. The postoperative course was uneventful, with no new ischemic lesions. Follow-up DSA at 6 and 12 months demonstrated nearly complete occlusion of the VADA (OKM grade C3) with preserved branch flow (Figure 2H).

## Discussion

### Natural course of VADA with ischemic presentation

Intracranial arterial dissections most frequently involve the vertebral artery (≈82%), and ischemic onset is relatively common (≈33%) (3). Vertebral artery dissections often stabilize as the arterial lumen gradually normalizes, typically within three months. However, a subset of cases demonstrates persistent or progressive aneurysmal dilatation (1, 4, 5). Yoshimoto and Wakai reported that 4 of 13 lesions without stenosis remained aneurysmal (5). Similarly, Kobayashi found that although 80.5% of VADA lesions showed no morphological deterioration, lesions measuring ≥10 mm tended to enlarge, emphasizing the need for short-interval imaging when large aneurysmal sacs are present (2).

When focusing specifically on ischemia-onset VADA, Kai reported that 20 of 30 individuals (66%) experienced recurrent ischemic stroke and subsequently required surgical intervention (4).

The mechanism of ischemic stroke in VADA is heterogeneous and includes embolic infarction from a partially thrombosed aneurysm, thrombosis at a stenotic segment, and occlusion of perforating or branch arteries involved in the dissection.

In the present series, the presumed mechanism varied among cases, suggesting that ischemia-onset VADA should not be considered a uniform entity. Two cases were strongly suspected of embolism, while the other two were branch occlusion or embolism.

In our cohort, 2 of 4 individuals experienced recurrent ischemic events and ultimately underwent FD placement. In contrast, the incidence of aneurysm rupture after ischemic onset of VADA remains unclear. Sagoh et al. documented that aneurysm rupture often occurs when the dissection extends into the basilar artery, typically during the acute phase after an ischemic presentation (6).

In partially thrombosed VADA, which were frequent in our series, there is both a risk of hemorrhage and a substantial risk of recurrent embolic stroke originating from intra-aneurysmal thrombus. The natural history of partially thrombosed VADA is considered extremely poor, with multiple reports describing death or severe morbidity due to rupture, recurrent ischemia, or brainstem compression from mass effect (7–9).

In our series, no hemorrhagic events occurred after ischemic onset. However, 3 of 4 individuals demonstrated enlargement of the VADA, which we regarded as an increasing risk of rupture and therefore proceeded with surgical intervention.

### Treatment for VADA with ischemic onset

In VADA with ischemic onset, no definitive treatment strategy—medical or surgical—has yet been established. From a medical management standpoint, antithrombotic agents are often avoided in the acute phase because of concerns about hemorrhage. Although both antiplatelet and anticoagulant therapies have shown efficacy in extracranial artery dissection, anticoagulation for vertebral artery dissection extending intracranially has been associated with subarachnoid hemorrhage (10). Other reports have likewise described an unruptured VADA that subsequently developed subarachnoid hemorrhage after anticoagulation (2). Therefore, particular caution is required when initiating antithrombotic therapy during the acute phase.

When rupture occurs, enlargement persists, or ischemia recurs in ischemia-onset VADA, surgical intervention should be considered. Deconstructive therapy—including trapping with or without bypass and endovascular internal trapping—remains the most reliable method for preventing rupture and recurrent ischemic stroke. However, these approaches carry a substantial risk of ischemic complications due to occlusion of normal vessels (1, 11, 12). In particular, internal trapping with long embolization segments increases the likelihood of medullary infarction, which is associated with poor outcomes (13). Consequently, in ischemia-onset cases, deconstructive therapy may be excessively aggressive given its procedural risks.

Reconstructive approaches have recently emerged as important alternatives for VADA. These strategies, including stent-assisted coil embolization (SAC) and flow diversion (FD), preserve antegrade flow in the parent artery and are feasible regardless of contralateral vertebral artery status, thereby minimizing disruption of normal vascular anatomy. Consistent with our findings, Cerejo et al. reported the safety and efficacy of FD for VADA in both ischemic and hemorrhagic presentations (14). Comparative data evaluating SAC and FD for intracranial VADA indicate similar safety profiles but lower major recanalization rates with FD (15). Notably, SAC has been associated with higher retreatment rates in partially thrombosed aneurysms owing to recurrence (16). By contrast, FD has demonstrated complete occlusion rates of approximately 77.1%, with reported ischemic complication rates of 9.8% and mortality of 4.2%; however, greater intra-aneurysmal thrombus burden (>50%) has been linked to reduced likelihood of complete occlusion (17).

However, the therapeutic effect of flow diversion in ischemia-onset VADA may depend on the underlying mechanism of infarction. Flow diverter stents may reduce the risk of recurrent embolism by blocking thrombosis to outside of vessel lumen and promoting endothelialization. In addition, they may stabilize and improve flow dynamics in cases with stenosis-related thrombosis.

In contrast, in cases where infarction is caused by occlusion of perforating or branch arteries directly involved in the dissection, the preventive effect of flow diversion may be limited. Therefore, careful evaluation of the infarction mechanism is important when considering reconstructive treatment strategies in ischemia-onset VADA.

Especially in Cases 1 and 2, infarction occurred in the PICA or perforator territories arising from the dissected segment. The mechanism of infarction could not be definitively determined, both distal embolism and branch occlusion at the origin of the involved vessel related to the dissection were considered possible. In such cases, the potential benefit of flow diversion should be interpreted cautiously. In particular, when perforator involvement is suspected, endovascular reconstruction may theoretically carry a risk of ischemic complications due to branch compromise. However, unlike atherosclerotic plaque shift, perforator compromise in arterial dissection is more likely associated with dynamic changes in the dissected vessel wall and intramural hematoma, making the actual risk difficult to predict.

In our series, although complete occlusion (OKM D) was not achieved within 6–12 months, all individuals reached at least OKM C without peri-procedural stroke, and none experienced recurrent infarction during follow-up. These observations support FD as a reasonable reconstructive option for ischemia-onset VADA, including partially thrombosed aneurysms, in order to reduce the risks of aneurysm rupture and recurrent ischemic stroke.

### Timing and use of perioperative antiplatelet therapy

In intracranial arterial dissections with aneurysmal formation, the use of antithrombotic therapy in the acute phase is often approached cautiously because of the potential risk of hemorrhagic concern. However, the risk of hemorrhagic events and re-dissection is thought to decrease over time, and antiplatelet therapy may be acceptable in the subacute to chronic phase in selected patients.

In our series, two patients were already receiving antiplatelet therapy at the time of initial presentation—one due to a prior coronary stent and the other initiated at a referring institution for secondary prevention—and neither developed hemorrhagic complications during the observation period. Two patients underwent flow diversion in the early chronic phase (within 1 and 4 months after onset) because of aneurysmal enlargement. In these cases, dual antiplatelet therapy (DAPT) was initiated prior to the procedure under careful in-hospital monitoring, without hemorrhagic event.

Previous reports have also described the use of DAPT with loading prior to flow diverter deployment in unruptured VADA. (Oh, Front Neurol, 2022). Based on our experience and the available literature, initiation of DAPT after confirmation of radiological stability—such as resolution of acute ischemic lesions and absence of hemorrhagic changes—may be a reasonable strategy. In cases with rapid aneurysmal progression, earlier initiation under close monitoring may be justified. Overall, the timing of DAPT in ischemia-onset VADA should balance hemorrhagic risk and ischemic prevention and should be individualized based on imaging findings and clinical course.

Regarding postoperative antiplatelet therapy, prolongation beyond the standard regimen does not appear necessary solely due to ischemic presentation.

In our series, dual antiplatelet therapy was maintained for 6 months, followed by single antiplatelet therapy, with consideration of discontinuation after confirmation of complete occlusion (OKM grade D). No recurrent ischemic events were observed under this protocol, suggesting that conventional management strategies for flow diversion may be applicable to ischemia-onset VAD. However, close imaging follow-up is warranted to detect aneurysm growth or new ischemic events.

### Limitation

This study has several limitations. It represents a small, retrospective, single-center case series with short-term imaging follow-up; therefore, aneurysm occlusion and remodeling may continue to evolve beyond the observed period. Selection bias is unavoidable, as flow diversion was specifically performed in patients with aneurysmal enlargement or recurrent ischemic events.

In addition, the mechanism of infarction could not be definitively determined in all cases, and both embolic and branch-related processes may have contributed, which may affect interpretation of treatment effects.

Furthermore, the optimal timing of flow diversion after ischemic onset remains unclear, and the antiplatelet management strategy described in this study may not be generalizable to all patients.

Future multicenter studies focusing on ischemia-onset VADA are warranted to better define patient selection and treatment strategies.

## Conclusion

Flow diversion may be considered a reconstructive treatment option in selected cases of ischemia-onset VADA. It may provide protection against both recurrent embolic events and aneurysmal rupture within a single intervention, although further studies are required to confirm its safety and efficacy.

## Data Availability

The raw data supporting the conclusions of this article will be made available by the authors, without undue reservation.
